# Analysis of recurrence probability following radiotherapy in patients with CNS WHO grade 2 meningioma using integrated molecular-morphologic classification

**DOI:** 10.1093/noajnl/vdad059

**Published:** 2023-05-14

**Authors:** Maximilian Y Deng, Felix Hinz, Sybren L N Maas, Günes Anil, Philipp Sievers, Cristina Conde-Lopez, Jonathan Lischalk, Sophie Rauh, Tanja Eichkorn, Sebastian Regnery, Lukas Bauer, Thomas Held, Eva Meixner, Kristin Lang, Juliane Hörner-Rieber, Klaus Herfarth, David Jones, Stefan M Pfister, Christine Jungk, Andreas Unterberg, Wolfgang Wick, Andreas von Deimling, Jürgen Debus, Felix Sahm, Laila König

**Affiliations:** Department of Radiation Oncology, Heidelberg University Hospital, Heidelberg, Germany; Heidelberg Institute of Radiation Oncology (HIRO), Heidelberg, Germany; National Center for Tumor Diseases (NCT), Heidelberg, Germany; Heidelberg Ion-Beam Therapy Center (HIT), Department of Radiation Oncology, Heidelberg University Hospital, Heidelberg, Germany; Department of Neuropathology, Heidelberg University Hospital and CCU Neuropathology, German Consortium for Translational Cancer Research (DKTK), German Cancer Research Center (DKFZ), Heidelberg, Germany; Department of Neuropathology, Heidelberg University Hospital and CCU Neuropathology, German Consortium for Translational Cancer Research (DKTK), German Cancer Research Center (DKFZ), Heidelberg, Germany; Department of Pathology, University Medical Center Utrecht, Utrecht University, Utrecht, The Netherlands; Department of Radiation Oncology, Heidelberg University Hospital, Heidelberg, Germany; Heidelberg Institute of Radiation Oncology (HIRO), Heidelberg, Germany; National Center for Tumor Diseases (NCT), Heidelberg, Germany; Heidelberg Ion-Beam Therapy Center (HIT), Department of Radiation Oncology, Heidelberg University Hospital, Heidelberg, Germany; Department of Neuropathology, Heidelberg University Hospital and CCU Neuropathology, German Consortium for Translational Cancer Research (DKTK), German Cancer Research Center (DKFZ), Heidelberg, Germany; Division of Radiooncology-Radiobiology, German Cancer Research Center (DKFZ), Heidelberg, Germany; Department of Radiation Oncology, Perlmutter Cancer Center at New York University Langone Health at Long Island, New York, NY, USA; Department of Radiation Oncology, Heidelberg University Hospital, Heidelberg, Germany; Heidelberg Institute of Radiation Oncology (HIRO), Heidelberg, Germany; National Center for Tumor Diseases (NCT), Heidelberg, Germany; Heidelberg Ion-Beam Therapy Center (HIT), Department of Radiation Oncology, Heidelberg University Hospital, Heidelberg, Germany; Department of Radiation Oncology, Heidelberg University Hospital, Heidelberg, Germany; Heidelberg Institute of Radiation Oncology (HIRO), Heidelberg, Germany; National Center for Tumor Diseases (NCT), Heidelberg, Germany; Heidelberg Ion-Beam Therapy Center (HIT), Department of Radiation Oncology, Heidelberg University Hospital, Heidelberg, Germany; Department of Radiation Oncology, Heidelberg University Hospital, Heidelberg, Germany; Heidelberg Institute of Radiation Oncology (HIRO), Heidelberg, Germany; National Center for Tumor Diseases (NCT), Heidelberg, Germany; Heidelberg Ion-Beam Therapy Center (HIT), Department of Radiation Oncology, Heidelberg University Hospital, Heidelberg, Germany; Department of Radiation Oncology, Heidelberg University Hospital, Heidelberg, Germany; Heidelberg Institute of Radiation Oncology (HIRO), Heidelberg, Germany; National Center for Tumor Diseases (NCT), Heidelberg, Germany; Heidelberg Ion-Beam Therapy Center (HIT), Department of Radiation Oncology, Heidelberg University Hospital, Heidelberg, Germany; Department of Radiation Oncology, Heidelberg University Hospital, Heidelberg, Germany; Heidelberg Institute of Radiation Oncology (HIRO), Heidelberg, Germany; National Center for Tumor Diseases (NCT), Heidelberg, Germany; Heidelberg Ion-Beam Therapy Center (HIT), Department of Radiation Oncology, Heidelberg University Hospital, Heidelberg, Germany; Department of Radiation Oncology, Heidelberg University Hospital, Heidelberg, Germany; Heidelberg Institute of Radiation Oncology (HIRO), Heidelberg, Germany; National Center for Tumor Diseases (NCT), Heidelberg, Germany; Heidelberg Ion-Beam Therapy Center (HIT), Department of Radiation Oncology, Heidelberg University Hospital, Heidelberg, Germany; Department of Radiation Oncology, Heidelberg University Hospital, Heidelberg, Germany; Heidelberg Institute of Radiation Oncology (HIRO), Heidelberg, Germany; National Center for Tumor Diseases (NCT), Heidelberg, Germany; Heidelberg Ion-Beam Therapy Center (HIT), Department of Radiation Oncology, Heidelberg University Hospital, Heidelberg, Germany; Department of Radiation Oncology, Heidelberg University Hospital, Heidelberg, Germany; Heidelberg Institute of Radiation Oncology (HIRO), Heidelberg, Germany; National Center for Tumor Diseases (NCT), Heidelberg, Germany; Heidelberg Ion-Beam Therapy Center (HIT), Department of Radiation Oncology, Heidelberg University Hospital, Heidelberg, Germany; Clinical Cooperation Unit Radiation Oncology, German Cancer Research Center (DKFZ), Heidelberg, Germany; Department of Radiation Oncology, Heidelberg University Hospital, Heidelberg, Germany; Heidelberg Institute of Radiation Oncology (HIRO), Heidelberg, Germany; National Center for Tumor Diseases (NCT), Heidelberg, Germany; Heidelberg Ion-Beam Therapy Center (HIT), Department of Radiation Oncology, Heidelberg University Hospital, Heidelberg, Germany; Hopp Children’s Cancer Center Heidelberg (KiTZ), Heidelberg, Germany; Division of Pediatric Glioma Research, German Cancer Research Center (DKFZ), Heidelberg, Germany; Hopp Children’s Cancer Center Heidelberg (KiTZ), Heidelberg, Germany; Department of Pediatric Oncology, Hematology, Immunology and Pulmonology, University Hospital Heidelberg, Heidelberg, Germany; Division of Pediatric Neurooncology, German Cancer Consortium (DKTK), German Cancer Research Center (DKFZ), Heidelberg, Germany; Department of Neurosurgery, University Hospital Heidelberg, Heidelberg, Germany; Department of Neurosurgery, University Hospital Heidelberg, Heidelberg, Germany; Clinical Cooperation Unit Neurooncology, German Consortium for Translational Cancer Research (DKTK), German Cancer Research Center (DKFZ), Heidelberg, Germany; Department of Neurology, Heidelberg University Hospital, Heidelberg, Germany; Department of Neuropathology, Heidelberg University Hospital and CCU Neuropathology, German Consortium for Translational Cancer Research (DKTK), German Cancer Research Center (DKFZ), Heidelberg, Germany; Department of Radiation Oncology, Heidelberg University Hospital, Heidelberg, Germany; Heidelberg Institute of Radiation Oncology (HIRO), Heidelberg, Germany; National Center for Tumor Diseases (NCT), Heidelberg, Germany; Heidelberg Ion-Beam Therapy Center (HIT), Department of Radiation Oncology, Heidelberg University Hospital, Heidelberg, Germany; Clinical Cooperation Unit Radiation Oncology, German Cancer Research Center (DKFZ), Heidelberg, Germany; Department of Neuropathology, Heidelberg University Hospital and CCU Neuropathology, German Consortium for Translational Cancer Research (DKTK), German Cancer Research Center (DKFZ), Heidelberg, Germany; Hopp Children’s Cancer Center Heidelberg (KiTZ), Heidelberg, Germany; Department of Radiation Oncology, Heidelberg University Hospital, Heidelberg, Germany; Heidelberg Institute of Radiation Oncology (HIRO), Heidelberg, Germany; National Center for Tumor Diseases (NCT), Heidelberg, Germany; Heidelberg Ion-Beam Therapy Center (HIT), Department of Radiation Oncology, Heidelberg University Hospital, Heidelberg, Germany

**Keywords:** CNS WHO grade 2, CNV, DNA methylation profiling, meningioma, radiotherapy

## Abstract

**Background:**

The current World Health Organization (WHO) classification of brain tumors distinguishes 3 malignancy grades in meningiomas, with increasing risk of recurrence from CNS WHO grades 1 to 3. Radiotherapy is recommended by current EANO guidelines for patients not safely amenable to surgery or after incomplete resection in higher grades. Despite adequately predicting recurrence probability for the majority of CNS WHO grade 2 meningioma patients, a considerable subset of patients demonstrates an unexpectedly early tumor recurrence following radiotherapy.

**Methods:**

A retrospective cohort of 44 patients with CNS WHO grade 2 meningiomas were stratified into 3 risk groups (*low*, *intermediate*, and *high*) using an integrated morphological, CNV- and methylation family-based classification. Local progression-free survival (lPFS) following radiotherapy (RT) was analyzed and total dose of radiation was correlated with survival outcome. Radiotherapy treatment plans were correlated with follow-up images to characterize the pattern of relapse. Treatment toxicities were further assessed.

**Results:**

Risk stratification of CNS WHO grade 2 meningioma into integrated risk groups demonstrated a significant difference in 3-year lPFS following radiotherapy between the molecular *low-* and *high*-risk groups. Recurrence pattern analysis revealed that 87.5 % of initial relapses occurred within the RT planning target volume or resection cavity.

**Conclusions:**

Integrated risk scoring can identify CNS WHO grade 2 meningioma patients at risk or relapse and dissemination following radiotherapy. Therapeutic management of CNS WHO grade 2 meningiomas and future clinical trials should be adjusted according to the molecular risk-groups, and not rely on conventional CNS WHO grading alone.

**Key Points**

1. Integrated molecular-morphologic classification predicts recurrence probability in patients with central nervous system (CNS) World Health Organization (WHO) grade 2 meningiomas following radiotherapy.
2. Clinical management of CNS WHO grade 2 meningiomas should be adjusted according to molecular risk groups.

**Importance of the Study**

The study provides the largest cohort of CNS WHO grade 2 meningioma patients following radiotherapy with comprehensive treatment protocols, follow-up data (eg, treatment toxicities, pattern of recurrence) and integrated molecular data assembled to date. The study highlights that the predictive value of the integrated molecular-morphologic classification remains robust in a cohort of patients with CNS WHO grade 2 meningiomas following radiotherapy. Therapeutic management of CNS WHO grade 2 meningiomas and future clinical trials should be adjusted according to molecular risk groups.

Meningiomas represent the most common primary brain tumor in adults.^1^ The current, fifth edition of the World Health Organization (WHO) classification of tumors of the central nervous system (CNS) (2021) includes 3 grades in meningiomas, corresponding to the risk of recurrence following surgical resection.^1^ The current European Association of Neuro-Oncology (EANO) guideline recommends postoperative radiotherapy for patients who are not safely amenable to surgery after incomplete resection in higher grades (ie, grades 2–3) to reduce the risk of tumor progression or recurrence.^2^ In a large retrospective series of 7811 patients with CNS WHO grade 2 and 1936 patients with CNS WHO grade 3 meningiomas from the US National Cancer Database, who received surgical resection and/or radiotherapy from 2004 to 2014: 5-year overall survival (OS) rate was 75.9% in patients with grade 2, and 55.4% in patients with grade 3 meningiomas (*P* < .0001).^3^ Furthermore, gross total resection (GTR) and postsurgical fractionated radiotherapy (RT) were independent predictors of improved survival in patients with grade 2 meningiomas.^2,3^ In recent years, 2 prospective phase II trials have investigated the clinical outcome in “intermediate” and “high-risk” meningioma patients following surgical resection:

The NRG Oncology/RTOG 0539 trial by the Radiation Therapy Oncology Group (RTOG) treated 48 patients with “intermediate-risk” meningiomas, as defined by recurrent CNS WHO grade 1 or newly diagnosed CNS WHO grade 2 tumors after GTR, with either intensity-modulated or 3D-conformal RT with 54 Gy in 30 fractions.^4^ The estimated 3-year progression-free survival (PFS) was 93.8%, and overall survival was 96% for “intermediate-risk” meningiomas. For patients with “high-risk” meningiomas, as defined by new or recurrent CNS WHO grade 3 or recurrent CNS WHO grade 2 meningioma of any resection extent or newly diagnosed CNS WHO grade 2 tumors after subtotal resection, 3-year PFS was estimated at 58.8% with a local control of 68.9%, and an OS at 78.6% following intensity-modulated radiotherapy (IMRT) with 60 Gy in 30 fractions.^5^ The prospective EORTC 22042-26042 phase II trial by the European Organization for Research and Treatment of Cancer evaluated the clinical outcome following postoperative fractionated radiotherapy in 56 patients with newly diagnosed CNS WHO grade 2 meningioma and GTR.^6^ The 3-year PFS and OS were estimated at 88.7% and 98%, respectively.^6^ Notably, study patients of the EORTC 22042-26042 received a higher radiation dose (60 Gy in 30 fractions) compared with the RTOG trial (54 Gy in 30 fractions).^6^ Both US and European trials have illustrated a potential benefit of postoperative radiotherapy for patients with “intermediate and high-risk” meningiomas.

Despite adequately predicting survival outcomes for the majority of patients with CNS WHO grade 2 meningiomas, the conventional CNS WHO grading schema has certain limitations: a considerable number of patients demonstrate an unexpectedly early tumor recurrence, while other patients show long-term local tumor control.^1^ Previous studies on risk stratification based on molecular characteristics revealed that molecular grading seems to be more accurate in identifying patients at high risk of disease progression, especially for tumors at the interface of low to intermediate risk for progression, ie, histological CNS WHO grades 1–2.^7,8^ Specifically, a comprehensive epigenetic study utilized DNA methylation profiling to analyze 497 meningioma samples, identifying six distinct, clinically relevant methylation classes from 3 overarching methylation class families, associated with characteristic mutational, cytogenetic and gene expression patterns.^8^ Subsequently, an integrated molecular and morphologic risk scoring (IntS)—combining DNA methylation class, CNS WHO grading, and copy-number changes of chromosomal arms 1p, 6q, and/or 14q—was established to increase diagnostic accuracy, outperforming conventional CNS WHO grading.^7^

This present study investigates the predictive value of the integrated molecular-morphologic classification in a homogenously treated cohort of patients with CNS WHO grade 2 meningiomas following radiotherapy in terms of recurrence probability. The study aims to elucidate to which extent the differences in survival outcome can be attributed to the innate biological aggressiveness of the respective molecular meningioma subgroups in a homogenously treated cohort.

## Methods

### Patient Cohort and Clinical Characteristics

Patients with meningioma CNS WHO grade 2 were retrospectively assembled and clinical patient characteristics (eg, age at diagnosis, date of diagnosis, and sex), tumor characteristics (location, size, available histological [eg, brain invasion, mitosis per 10 consecutive high-power- fields of 0.16 mm^2^], and molecular features) and the course of treatment (including surgical resection, radiotherapy, and chemotherapy) were collected and obtained from the database of the University Hospital Heidelberg, the Heidelberg Institute for Radiation Oncology, and the NCT cancer registry. All study patients were irradiated at the Department of Radiation Oncology, University Hospital Heidelberg, within a timeframe between 2009 and 2022. The extent of tumor resection was assessed after neurosurgical procedures via postoperative MRIs according to current guidelines or surgical reports, and classified as GTR, subtotal resection (STR).^2,9–11^ In a subset of patients, where immediate postoperative MRIs and surgical reports were missing, pre-RT planning MRIs were evaluated to distinguish between GTR and macroscopic tumors. The study was approved by the Independent Ethics Committee of the Medical Faculty Heidelberg (S-293/2022).

### DNA Methylation Profiling of Meningiomas and Integrated Model Score

DNA was extracted from FFPE tumor material using a Maxwell system (Promega, Fitchburg, WI, USA) and the Maxwell 16 FFPE Plus LEV DNA Purification Kit, according to the manufacturer’s guidelines. DNA concentration was determined via Invitrogen Qubit dsDNA BR Assay Kit (Thermo Fisher Scientific, Waltham, MA, USA) and FLUOstar Omega Microplate Reader (BMG Labtech GmbH, Ortenberg, Germany). Genome-wide DNA methylation profiles were previously generated using the Illumina Infinium HumanMethylation450 (450k) and MethylationEPIC (EPIC) array according to the manufacturer’s guidelines (Illumina, San Diego, USA) at the Genomics and Proteomics Core Facility of the DKFZ (Heidelberg, Germany), according to the manufacturer’s guidelines (Illumina, San Diego, USA). All computational analyses were performed in R version 3.4.1 (R Development Core Team, 2018), as previously described.^7,8,12^ Meningioma methylation class families were determined by the highest scoring family score as obtained from the v12.5 DKFZ brain tumor classifier at www.molecularneuropathology.org. The meningioma methylation classes—as initially presented in Sahm et al.^8^—were applied in the study. Reference samples were chosen by selecting cases of the respective group without deviation from the average copy-number and mutational aberrations of the group. In brief, methylation data points were assessed through fluorescent signals from the methylated and unmethylated alleles, which were used to calculate the beta-value, ranging from 0 (non-methylated locus) to 1 (methylated locus). Raw signal intensities were retrieved from IDAT-files using the minfi Bioconductor package version 1.24.0.^13^ EPIC and 450k samples were merged to a combined data set through the selection of probes, which were represented on both arrays (combineArrays function, minfi). Each sample was individually normalized using background correction by shifting the fifth percentile of negative control probe intensities to 0. A dye-bias correction was performed, where the mean of normalization control probe intensities was scaled to 10 000 for both color channels. A correction for the type of material tissue (FFPE or frozen) and array (450k or EPIC) was conducted by fitting univariate, linear models to the log2-transformed intensity values (removeBatchEffect function, limma package version 3.34.5) to avoid potential batch effects. Beta-values were generated using the retransformed intensities with an offset of 100, as suggested by Illumina. The following filtering criteria were applied before further analysis: Removal of probes targeting the X and Y chromosomes, removal of probes that contained a single-nucleotide polymorphism (dbSNP132 Common) at the targeted CpG-site or 5 base pairs before and after the respective CpG-site, and probes which were included in only one of the arrays. Further probes, which could not be mapped uniquely to the human reference genome (hg19), allowing for one mismatch only, were also removed. The 1-variance weighted Pearson correlation between the samples was calculated, generating a distance matrix. This matrix was utilized as the input for t-distributed stochastic neighbor embedding (t-SNE) analysis (Rtsne package version 0.13) with the application of the following non-default parameters: Theta = 0, pca = F, max_iter = 2500, perplexity = 20. The 10 000 probes with the highest standard deviation were selected to calculate the Euclidean distance between the samples. Unsupervised hierarchical clustering was performed using Ward's linkage method for sample clustering. Represented probes were reordered by complete linkage hierarchical clustering of the Euclidean distance between the probes.^8,12^ copy-number variation analysis from 450k and EPIC methylation array data were performed using the conumee Bioconductor package version 1.12.0. Furthermore, Integrated Model Scores (IMS) were calculated by summarizing the respective scores of methylation family (range: 0–4), CNS WHO grading (range: 0–2) and chromosomal losses of 1p, 6q, and/or 14q (range: 0–3).^7^ IMS were defined as: Low (0–2), intermediate (3–5), and high (>5), as previously described.^7^ A subset of study patients were previously incorporated in generating the meningioma methylation classes (*13*/44)^8^ and IMS (*17*/44).^7^

### Planning and Treatment Features

Patients were immobilized with custom thermoplastic masks and treatment planning simulation scans (eg, computed tomography and cranial MRI [cMRI]). Gross tumor volume included the macroscopic tumor and/or resection cavity. A safety margin was set for suspected microscopic tumor spread to define the clinical target volume, while respecting anatomic boundaries. An isotropic margin of 3–5 mm was used for creation of the planning target volume (PTV) to account for geometric uncertainties and physical inaccuracies of the RT technique. Treatment planning followed the principle of as low as reasonably achievable (ALARA) and was according to the constraints of ICRU reports 50 and 62, and normal tissue constraints according to QUANTEC and Emami et al.^14^ Radiotherapy was administered in 1.8–3.0 Gy single doses over 5–6 fractions per week. Photon radiotherapy was administered with 3D-conformal radiotherapy (3DCRT) or intensity-modulated radiotherapy (IMRT). For treatment standardization, radiation dose equivalents in 2 Gy fractions (EQD2) and relative biological effectiveness (RBE)-weighted doses for proton beam irradiation were calculated. For proton beam irradiation, RBE was estimated to be 1.1 according to the current clinical standards. For carbon-ion irradiation, biological dose was calculated using local effect model I (LEM I) with an alpha/beta ratio of 2.

### Survival Analysis, Toxicity Analysis, and Statistical Considerations

Local PFS (l-PFS) following surgical resection was selected as primary endpoint. L-PFS was defined from the surgical resection (or radiotherapy in case of salvage RT) until tumor progression within the surgical cavity and the PTV of the radiation plan. Tumor progression was defined according to the criteria presented by the Response Assessment in Neuro-Oncology Working Group (RANO).^15^ Radiation treatment plans were correlated with available clinical follow-up MR imaging to evaluate the pattern of relapse (infield vs. outfield). Overall survival was determined from the date of initial diagnosis until death. Patients, who became lost to follow-up were censored at the date of the last follow-up to define overall and PFS. Overall survival (OS) and PFS were estimated via Kaplan–Meier analysis.

Treatment toxicity was assessed and classified according to the NCI Common Terminology Criteria for Adverse Events (CTCAE) version 5.0 8–12 weeks after RT (acute toxicity) or at the last follow-up (late toxicity) (http://ctep.cancer.gov/protocolDevelopment/electronic_applications/ctc.htm).^16^*P*-value < .05 was considered significant. Radiation-induced contrast enhancement (RICE) was defined as new post-treatment contrast enhancement on cMRI in the surrounding brain tissue within the 80% isodose line analogous to RANO criteria during the follow-up period.^17^

## Results

### Clinical Patient Characteristics and Treatment Regimes

**Figure 1.**
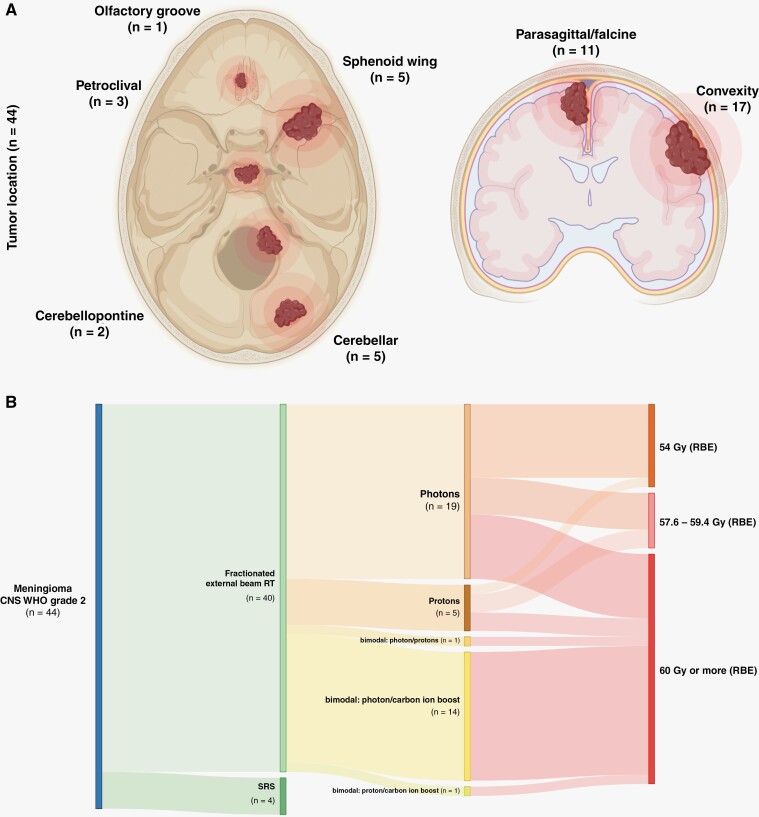
Tumor location and postoperative radiotherapeutic concepts. (A) Tumor location are shown, with numbers in brackets indicating group size. (B) Fractionated external beam radiotherapy (RT) was applied in 40 patients with central nervous system World Health Organization grade 2 meningiomas, and stereotactic radiosurgery (SRS) in 4 patients. A diverse spectrum of modalities was utilized, encompassing photons, protons and bimodal concepts with protons and/or carbon-ions. RBE, relative biological effectiveness.

The study cohort comprised 44 patients with CNS WHO grade 2 meningiomas, with a median age at diagnosis of 49.5 years (range: 15–75 years). Female patients (*n* = 24) were slightly overrepresented in our cohort over males (*n* = 20). Meningiomas were located along the convexity (*n* = 17), parasagittal/falcine (*n* = 11), at the sphenoid wing (*n* = 5), cerebellar (*n* = 5), and cerebellopontine (*n* = 2), petroclival (*n* = 3) and in the olfactory groove (*n* = 1) (Figure 1A).

Macroscopic (residual) tumor in the pre-RT planning MRI was demonstrated in 37/44 patients, while GTR was reached in 7/44 patients. All patients received immediate postoperative radiotherapy following surgical resection, either via fractionated external beam RT (*n* = 40) or stereotactic radiosurgery (*n* = 4). Fractionated external beam RT was applied with a median dose of 60 Gy (range: 54–68 Gy), comprising conventional photon RT (3D-conformal or intensity-modulated) (*n* = 19), proton RT (*n* = 5) and carbon-ion RT in combination with photon RT (*n* = 14). Furthermore, bimodal concepts using photons and protons, and protons and carbon-ion RT were selected in one patient, respectively (Figure 1B). Additional clinical patient characteristics and treatment details are listed in Table 1.

**Table 1.** Clinical Patient Characteristics and Radiotherapy Concepts

Patients	Overall Cohort
*Gender (n = 44)*	
Female	24 [54.5]
Male	20 [45.5]
*Age at initial diagnosis (n = 44)*	
Median	49.5
Minimum–maximum	15–75
*Extent of resection (n = 44)*	
Gross total resection	7 [15.9]
Subtotal resection/macroscopic tumor (residue)	37 [84.1]
*Total dose in Gy/ Gy (RBE)*	
Median	60
Minimum–maximum	54.0–68
Single dose	1.8–3.0
*RT modality (n = 44)*	
Photon (3D-conformal, IMRT)	19 [43.2]
Protons	5 [11.4]
Bimodal: photons and protons	1 [2.3]
Bimodal: photons and C12-ion	14 [31.8]
Bimodal: protons and C12-ion	1 [2.3]
Stereotactic radiosurgery	4 [9.1]
*DNA methylation class (n = 44)*	
Benign	17 [38.6]
Intermediate	22 [50.0]
Malignant	5 [11.4]
*Copy-number changes (n = 44)*	
1q loss	29 [65.9]
6q loss	22 [50.0]
14q loss	21 [48.7]
*Integrated risk scoring (IntS) (n = 44)*	
Low	9 [20.5]
Intermediate	19 [43.2]
High	16 [36.4]

If not otherwise visible, absolute and relative frequencies were shown. Relative frequencies are based on the available data and exclude missings.

Gy RBE, Gray Relative Biological Effectiveness.

### Treatment Toxicities

No acute treatment toxicities exceeding CTCAE grade 2 were observed during radiotherapy or within 3 months after RT completion. Late toxicities (>3 months after RT) exceeding CTCAE grade 3 were observed in 13.6% (6/44) following radiotherapy, while sensory and motor deficits were often associated with symptomatic radiation-induced contrast enhancement (Table 2). No association between the occurrence of RICE and molecular risk group was observed.

**Table 2.** Treatment Toxicity

Toxicity	Event/ Overall Cohort [%]	Mild Acute Events [%] (CTCAE 1-2°)	Severe Acute Events [%] (CTCAE 3-4°)	Mild Chronic Events [%] (CTCAE 1-2°)	Severe Chronic Events [%] (CTCAE 3-4°)
Radiation-induced contrast enhancement	**22.7** (10/44)	-	—	**15.9** (7/44)	**6.8** (3/44)
Fatigue	**63.6** (28/44)	**25.0** (11/44)	—	**45.5** (20/44)	—
Headache	**43.2** (19/44)	**29.5** (13/44)	—	**34.1** (15/44)	—
Dizziness	**29.5** (13/44)	**22.7** (10/44)	—	**20.5** (9/44)	—
Hypopituitarism	—	—	—	—	—
Nausea	**13.6** (6/44)	**11.4** (5/44)	—	**4.5** (2/44)	—
Motor deficits	**13.6** (6/44)	—	—	**9.1** (4/44)	**6.8** (3/44)
Sensory deficits	**34.1** (15/44)	—	—	**31.8** (14/44)	**2.3** ^(a)^ (1/44)
Seizures	**9.1** (4/44)	**4.5** (2/44)	**2.3** (1/44)	**6.8** (3/44)	—
Focal alopecia	**70.5** (31/44)	**18.2** (8/44)	—	**70.5** (31/44)	—

^(a)^ A decrease in visual acuity (CTCAE grade 3) was expected in one patient based on the radiation treatment plan; patient informed consent was obtained.

CTCAE: NCI Common Terminology Criteria for Adverse Events.

RICE were encountered in 22.7% (10/44), mostly frequently following bimodal radiotherapy with a carbon-ion boost with 18 Gy (RBE) in single doses of 3 Gy (RBE) in combination with photon radiotherapy with 50 Gy in single doses of 2 Gy (6/44). Asymptomatic patients were closely observed during follow-up (4/44), while patients presenting clinical symptoms (6/44) received high-dose dexamethasone. Full regredience of RICE was achieved in 3 patients after dexamethasone. However, vascular endothelial growth factor (VEGF) inhibition using bevacizumab was required in 3 patients due to further progression of the RICE, thus, qualifying as radiation necrosis (CTCAE > grade 3). Further toxicities are listed in Table 2.

### Integrated Molecular-Morphologic Scoring Predicts Local Recurrence Probability in CNS WHO Grade 2 Meningiomas

**Figure 2.**
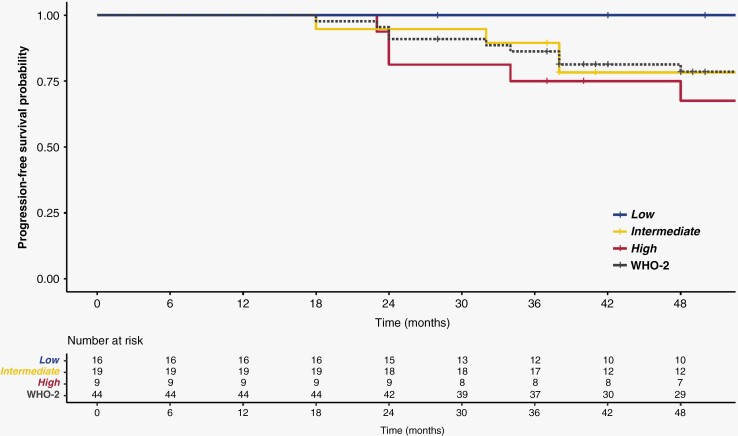
Integrated molecular-morphologic classification predict recurrence probability in patients with CNS WHO grade 2 meningiomas following radiotherapy. Kaplan–Meier curve for progression-free survival (PFS) revealed that the integrated risk groups (*low, int, and high*) correlate with distinct clinical outcome. Dotted line indicates the entire patient cohort with CNS WHO grade 2 meningioma. Local PFS after 3 years following radiotherapy was statistically significant between the *low-* and *high*-risk patients (*P* = .0048).

Median follow-up time was 78 months (range: 28–258 months). Local PFS (3y-lPFS) was estimated at 86.2% (95% CI: 76.6%–98.5%) after 3 years for the entire study cohort (Figure 2).

DNA methylation-based classification (v12.5 DKFZ brain tumor classifier) allotted 17 patients (38.6%) to the *benign*-, 22 (50.0%) to the *intermediate*-, and 5 patients (11.4%) to the *malignant* methylation class family. Chromosomal copy-number changes were frequently encountered, with loss of chromosomal arm 1q in 65.9% (29/44), loss of 6q in 50% (22/44), and loss of 14q in 48.7% (21/44). All risk parameters (CNS WHO grade 2, CNVs, DNA methylation family) were merged into the three-tiered integrated molecular-morphologic score (*low*, *intermediate*, and *high*).^7^ Risk stratification into the integrated molecular-morphologic risk groups displayed substantial differences in 3-year lPFS: with 100% in the *low*-risk class (*n* = 9), 89.5% in the *intermediate* (95% CI: 76.7%–100%, *n* = 19) and 75.5% in the *high*-risk class (95% CI: 56.5%–99.5%, *n* = 16; *P* = .0048 compared to the *low*-risk class) (Figure 2). Differences in local tumor control in CNS WHO grade 2 meningiomas following radiotherapy were significant between the *low-* and *high-*risk methylation class (*P* = .0048), while conventional histological markers (eg, histological brain invasion [univariate analysis: HR = 0.47, CI: 0.16–1.42, *P* = .18] or mitosis per 10 high-power-field [univariate analysis: HR = 1.01, CI: 0.89–1.15, *P* = .86]) were unable to stratify the study cohort into prognostically significant subgroups (Supplementary Figure 1).

Subsequent recurrence pattern analysis revealed that 87.5% of initial relapses occurred within the planning (RT) target volume (PTV) or resection cavity (*n* = 21/24). Distant, out-of-field recurrences were encountered—at the time of initial relapse—in 3 patients with meningiomas of the *high*-risk group. Notably, 7 out of 35 patients with *intermediate or high*-risk meningiomas presented distant recurrences during the course of disease, often with multifocal presentation (eg, spinal cord).

## Discussion

Following subtotal resection of CNS WHO grade 2 meningiomas, postoperative radiotherapy was strongly recommended by the current EANO guideline.^2^ However, the benefit of fractionated RT after GTR remains unclear, only allowing a level IV evidence to the question of whether CNS WHO grade 2 meningiomas should be irradiated.^1,2^

In recent years, DNA methylation-based brain tumor classification was demonstrated to represent a robust diagnostic tool in predicting tumor recurrence.^7,8^ However, comprehensive treatment protocols and follow-up data (eg, treatment toxicities, pattern of recurrence) were absent in these large-scale epigenomic studies. Thus, a potential therapeutic bias between the respective methylation classes of meningiomas (eg, adjuvant treatment vs. watch-and-wait) cannot be excluded with certainty. With the addition of comprehensive treatment and follow-up data, this present study demonstrates that the predictive value of the previously presented integrated morphologic, copy-number variation- and methylation family-based meningioma classifier remains robust in a cohort of patients with CNS WHO grade 2 meningiomas following radiotherapy.^7^ Local PFS after 3 years was estimated at 86.2% for the entire study cohort of CNS WHO grade 2 meningiomas. The survival outcome of our study cohort lies between the reported outcomes of the “high-risk” (3-year PFS: 58.8%) and “intermediate-risk” (3-year PFS: 96%) group of the NRG Oncology/RTOG 0539 trial, as defined by CNS WHO grading and extent of resection alone. Our study provides substantial evidence that molecular classification predicts recurrence probability in patients with CNS WHO grade 2 meningiomas, with a 3-year lPFS of 75.5% in the *high-* and 89.5% in the *intermediate*-risk group. This study supports the notion that the difference in survival outcome is largely attributed to the distinct, innate biological behavior of the respective molecular meningiomas classes within CNS WHO grade 2 meningiomas. While previous reports provided evidence for the prognostic value of the extent of resection, there was no significant difference between subtotal/macroscopic (residual) tumor and GTR in terms of local tumor control in our cohort, which could, however, likely be attributed to the limited cohort size.^2,18,19^

Previous studies have reported that a total dose of 60 Gy was insufficient in achieving long-term tumor control in CNS WHO grade 2–3 meningiomas. Thus, dose escalation exceeding 60 Gy may represent a potential strategy to increase local tumor control.^20–22^ Overall, the selection of different treatment concepts (eg, RT modality, dosage) based on individual clinical indications, may represent a limitation of our study.

RICE were encountered in 22.7% during follow-up, while most patients only presented mild neurological symptoms (CTCAE grade 1–2). Only 3/44 patients required high-dose dexamethasone and subsequent anti-VEGF inhibition using bevacizumab due to persisting neurological symptoms. The rate of radiation-induced contrast enhancement in this study was lower compared to previous reports on adult patients with low-grade gliomas.^23^ Late toxicities of grade 3 or more were encountered in 13.6 % (6/44) in our study cohort, comparable with previous reports of the EORTC 22042-26042 phase II study with 14%.^6^

Therapeutic management in molecular *high*-risk meningiomas may be adjusted according to the recommendations for conventional CNS WHO grade 3 meningiomas due to their similar clinical course (eg, RT-boost for the macroscopic residual tumor).^1,2,5^ On the contrary, the omission of postoperative radiotherapy may be discussed for CNS WHO grade 2 meningioma patients with a molecular *low*-risk profile and the absence of other potential risk factors (eg, GTR). The discrepancy between histological grading and molecular risk group strongly indicates that future studies with matched-pair analyses and a non-irradiated, observation-only cohort with available molecular characterization are required to elucidate the exact benefit of postoperative RT for the different constellations of CNS WHO grading (grade 1–3) and molecular risk group, as well as resection status (STR vs. GTR). In particular, the analysis of patients with CNS WHO grade 1 meningiomas with *intermediate*- or *high*-risk profiles are strongly warranted to identify patients at risk who might benefit from postoperative radiotherapy.

Furthermore, current EANO guidelines recommend a follow-up interval of 6 months in the initial 5 years after initial diagnosis for CNS WHO grade 2 meningiomas, without recognizing the molecular meningioma class (or other molecular markers) during follow-up management. Our study provides evidence that follow-up intervals may be adjusted according to the molecular risk groups, eg, every 3 months for *high*-risk CNS WHO grade 2 meningiomas.

Integrated molecular-morphologic classification of CNS WHO grade 2 meningiomas provides a powerful tool in identifying patients at risk who remained unrecognized based on histology-based WHO grading. Therapeutic management of CNS WHO grade 2 meningiomas and future clinical trials should be adjusted according to the molecular risk groups, and not rely on conventional CNS WHO grading alone.

## Supplementary Material

vdad059_suppl_Supplementary_Figure_S1

vdad059_suppl_Supplementary_Table_S1
