# Supplementary material for: Analysis of recurrence probability following radiotherapy in patients with CNS WHO grade 2 meningioma using integrated molecular-morphologic classification
Source: Neurooncol Adv. 2023 May 14;5(1):vdad059. doi: 10.1093/noajnl/vdad059 (PMC10246580; doi:10.1093/noajnl/vdad059)

**Supplementary Figure 1.**

Suppl. Figure 1. Univariate (A) and multivariate analysis (B) of progression-free survival.


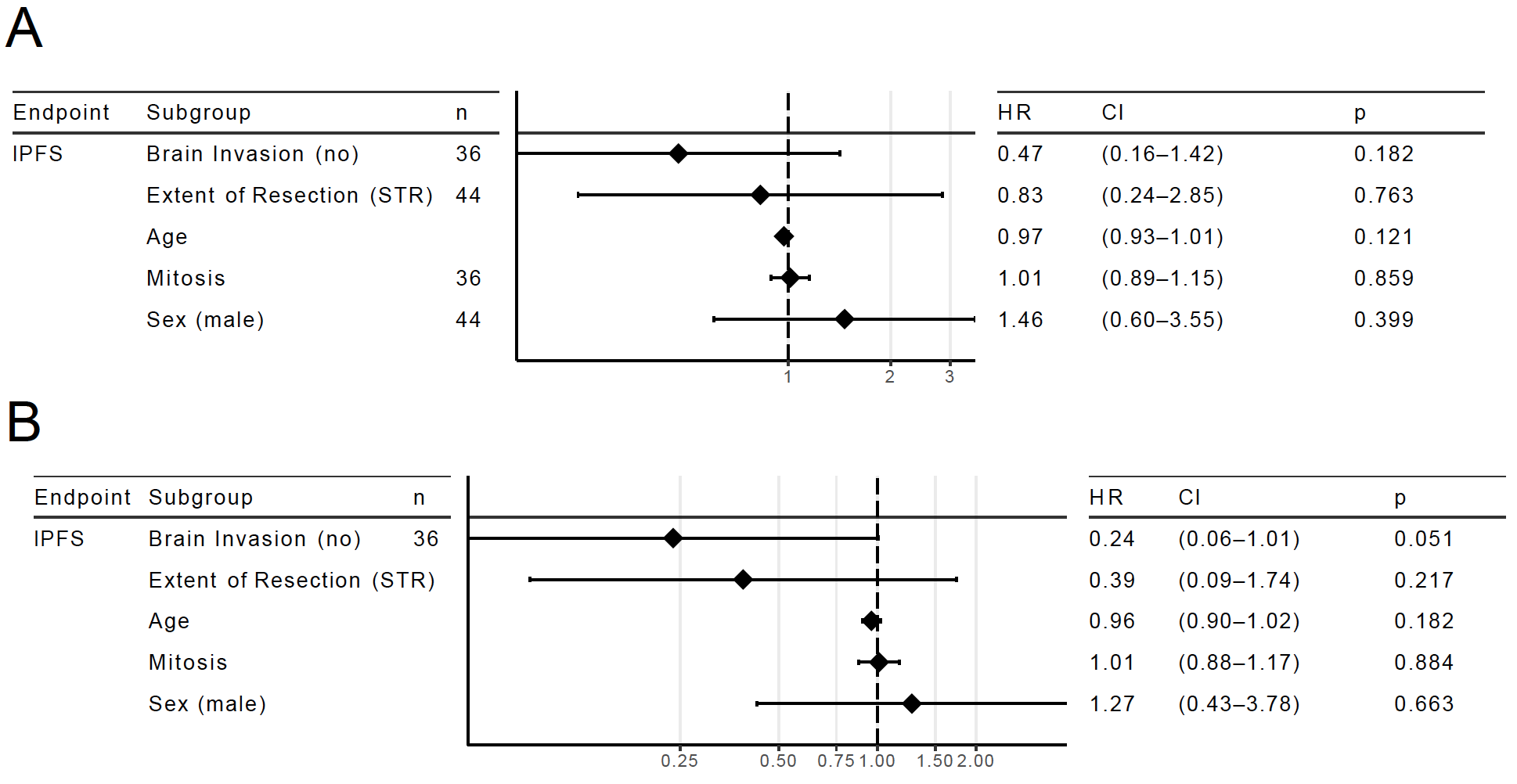

Supplement: vdad059_suppl_Supplementary_Figure_S1 [file vdad059_suppl_supplementary_figure_s1.docx]
