# Supplementary material for: Analysis of recurrence probability following radiotherapy in patients with CNS WHO grade 2 meningioma using integrated molecular-morphologic classification
Source: Neurooncol Adv. 2023 May 14;5(1):vdad059. doi: 10.1093/noajnl/vdad059 (PMC10246580; doi:10.1093/noajnl/vdad059)
Supplement: vdad059_suppl_Supplementary_Table_S1 [file vdad059_suppl_supplementary_table_s1.docx]

**Supplementary Table 1**

| ID | Included.in.Sahm.et.al..(2017) | Included.in.Maas.et.al..(2021) |
| --- | --- | --- |
| RT-Men01 | yes | yes |
| RT-Men02 | no | no |
| RT-Men03 | no | no |
| RT-Men04 | no | no |
| RT-Men05 | no | no |
| RT-Men06 | no | no |
| RT-Men07 | no | yes |
| RT-Men08 | no | yes |
| RT-Men09 | yes | no |
| RT-Men10 | no | no |
| RT-Men11 | no | no |
| RT-Men12 | yes | no |
| RT-Men13 | no | no |
| RT-Men14 | no | no |
| RT-Men15 | no | no |
| RT-Men16 | no | no |
| RT-Men17 | no | no |
| RT-Men18 | no | no |
| RT-Men19 | no | no |
| RT-Men20 | yes | yes |
| RT-Men21 | yes | yes |
| RT-Men22 | no | yes |
| RT-Men23 | yes | yes |
| RT-Men24 | no | yes |
| RT-Men25 | no | no |
| RT-Men26 | no | no |
| RT-Men27 | no | no |
| RT-Men28 | no | yes |
| RT-Men29 | no | no |
| RT-Men30 | yes | yes |
| RT-Men31 | yes | yes |
| RT-Men32 | yes | yes |
| RT-Men33 | yes | no |
| RT-Men34 | yes | yes |
| RT-Men35 | no | yes |
| RT-Men36 | no | yes |
| RT-Men37 | no | no |
| RT-Men38 | no | no |
| RT-Men39 | yes | yes |
| RT-Men40 | no | no |
| RT-Men41 | yes | no |
| RT-Men42 | no | no |
| RT-Men43 | no | no |
| RT-Men44 | no | yes |
